# New evidence for the effect of type 2 diabetes and glycemic traits on lung function: a Mendelian randomization and mediation analysis

**DOI:** 10.1016/j.clinsp.2025.100693

**Published:** 2025-07-15

**Authors:** Shuo Xie, Liping Yu, Lulu Song, Fei Chen, Wanlu Ma, Yifan He, Xiaoping Chen, Ying Yang, Bo Zhang

**Affiliations:** a Department of Endocrinology, China-Japan Friendship Hospital, Beijing, China; b Chinese Academy of Medical Sciences & Peking Union Medical College, Beijing, China

**Keywords:** Lung Function, Type 2 Diabetes, Systolic Blood Pressure, Smoking;Mendelian Randomization, Genome-Wide Association Study

## Abstract

**Background:**

Type 2 Diabetes Mellitus (T2DM) is common worldwide. Patients with T2DM often experience reduced lung function, but the relationship between the two conditions remains uncertain. This study employed Mendelian Randomization (MR) to explore the causal link between T2DM and lung function, and to identify the factors that mediate this relationship.

**Methods:**

Genome-wide association study data were obtained from public databases. Four glycemic traits were considered exposures, whereas lung function was considered an outcome. Inverse Variance Weighting (IVW) was used to investigate causality, supplemented by MR-Egger and weighted medians methods. Multivariable MR (MVMR) analysis investigated whether T2DM had an independent impact on lung function. Mediation analysis was employed to examine potential mediating impacts.

**Results:**

IVW revealed that T2DM was associated with decreased forced expiratory volume in 1s (FEV1) (p = 0.012) and Forced Vital Capacity (FVC) (p = 0.004). After adjusting for potential confounders, the effects of T2DM on FEV1 and FVC remained significant. Mediation analysis demonstrated that smoking and Systolic Blood Pressure (SBP) partially mediated the causal relationship between T2DM, and FEV1 and FVC. The proportions mediated by smoking, SBP, and both factors on FEV1 were 0.302, 0.412, and 0.418, respectively, while the proportion mediated by SBP on FVC was 0.594.

**Conclusions:**

This study validated the causal relationship between T2DM and decreased lung function, with smoking and SBP acting as mediators. This study provides a novel perspective on the occurrence and development mechanisms of reduced lung function as well as potential new targets for metabolic intervention in treatment.

## Highlights

• Type 2 diabetes linked to reduced lung function, with smoking and high BP as mediators.

## Introduction

Diabetes mellitus is one of the most prevalent global epidemics, classified as a group of diseases characterized by chronic hyperglycemia.^1^ The most common forms are Type 2 Diabetes Mellitus (T2DM), type 1 Diabetes Mellitus (T1DM), and gestational diabetes mellitus,^2^ with T2DM being the predominant contributor to the current global epidemic.^3^ Between 1980 and 2014, the number of adults diagnosed with diabetes increased from 108 million to 422 million,^4^ with type 2 diabetes accounting for more than 90% of these cases.^5^ The global prevalence of diabetes is estimated to reach 700 million by 2045.^6^ Diabetes ranks among the top three diseases worldwide, and its prevalence is increasing.^7^ Furthermore, diabetes is linked to an increased cost of healthcare, which is estimated to be $850 billion worldwide.^8^ It is an ideal target for prevention owing to its enormous cost.

Genetic and environmental factors are associated with decreased lung function. Numerous epidemiological studies have indicated that individuals with T2DM tend to exhibit reduced lung function compared with those without diabetes.^9^^,^^10^ The variations in findings in previous studies could be attributed to biases or confounding factors present in observational epidemiological studies, including limited sample sizes, differences in demographic characteristics, reverse causation, and selection bias. T2DM is a persistent metabolic condition that progresses slowly and initially has hidden symptoms, making the connection between T2DM and lung function unclear.^11^

Mendelian Randomization (MR) is an analytical method used to estimate the causal impact of outcomes of interest by utilizing genetic variants associated with exposure.^12^ MR is more effective than traditional observational methods for preventing confounders and reverse causality.^13^ An MR study was conducted to gain new insights into the pathogenesis of lung function impairment. Multivariable MR (MVMR) was used to adjust for potential confounders that might affect causal estimation, whereas mediation analysis was used to explore the potential mediating role of smoking and Systolic Blood Pressure (SBP) in the causal link between T2DM and lung function. The present study offers a new outlook for investigating the mechanisms underlying the onset and progression of lung dysfunction and identifying new targets for metabolic therapy interventions.

## Materials and methods

### Reporting guidance

This study was conducted following the Strengthening the Reporting of Observational Studies in Epidemiology using the MR (STROBE-MR) guideline.^14^

### Study design

**Fig. 1:**
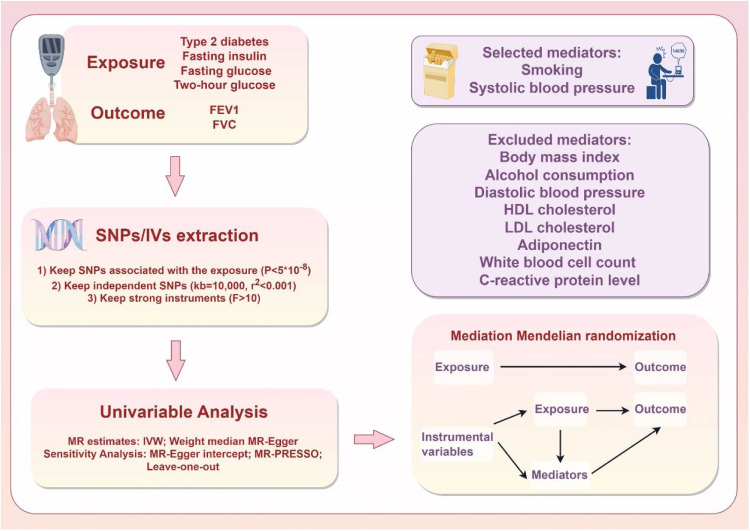
Study overview. FEV1, Forced Expiratory Volume in 1s; FVC, Forced Vital Capacity; SNPs, Single Nucleotide Polymorphisms; MR, Mendelian Randomization; IVW, Inverse Variance Weighting; IV, Instrumental Variable.

MR and mediation analyses were used to determine the causal connection between glycemic characteristics and lung function and to explore potential mediators of this relationship. As shown in Fig. 1 MR analysis relies on three important assumptions. The overall causative association between glycemic traits and lung function was first investigated, followed by consideration of the ratio of mediating factors to causation. The MR investigation used publicly available Genome-Wide Association Study (GWAS) datasets. For the original GWAS, each participant had provided written informed consent.

### Data sources

Glycemic traits include T2DM, hyperinsulinemia, and hyperglycemia.^15^ Data on glycemic traits (T2DM, fasting insulin, fasting glucose, and two-hour glucose) from GWAS summary datasets were acquired from the Medical Research Council Integrative Epidemiology Unit OpenGWAS database at https//gwas.mrcieu.ac.uk/. This study examined four factors from various GWAS; T2DM (dataset finn-b-E4_DM2; n=215,654), fasting insulin (ieu-b-116; n = 108,557), fasting glucose (ieu-b-114; n=133,010), and two-hour glucose (ebi-a-GCST90002227; n = 63,396). Additional details of each characteristic are provided in Supplemental Table 1.

Data on lung function, specifically Forced Expiratory Volume in 1s (FEV1) (ukb-b-19657) and Forced Vital Capacity (FVC) (ukb-b-7953), collected by the Medical Research Council Integrative Epidemiology Unit were utilized as primary measures, involving a cohort of 421,986 individuals of European descent. For the MR analysis, genetic variants were chosen that met the criteria for genome-wide significance (p < 5 × 10^−8^).

### Informed consent statement and ethics approval statement

Analyses were conducted using publicly accessible datasets from published GWAS or consortia without the need for additional consent or ethical approval for the current study.

### Genetic instrumental variable (IV) selection

To identify Single Nucleotide Polymorphisms (SNPs) that were strongly associated with exposure or outcome, a threshold for genome-wide significance of p < 5 × 10^−8^ was established. To avoid bias caused by Linkage Disequilibrium (LD), SNPs with R^2 ^ < 0.001 and kb>10,000 which were strongly associated with exposure were selected.^16^ Palindromic SNPs with mid-range allele frequencies were excluded. Furthermore, the F-statistic was calculated as F=β2exposure/SE2exposure to measure the effectiveness of the genetic instrument for all SNPs, with SNPs having an F-value <10 being classified as weak instruments.^17^^,^^18^

To avoid possible pleiotropic effects, SNPs were additionally examined using PhenoScanner V2 (http://www.phenoscanner.medschl.cam.ac.uk/) to determine whether instrumental variables were linked to confounders or risk factors.^19^^,^^20^

### Mendelian randomization analysis

The main method used in MR to estimate the causal relationship between T2DM and glycemic traits with lung function was Inverse-Variance Weighting (IVW).^21^ Due to the variability in the databases utilized, a random-effects model was used for all MR analyses. Concurrently, MR-Egger and MR-PRESSO were used to conduct sensitivity tests to assess the reliability of the findings and confirm the presence of horizontal pleiotropy. The MR-Egger regression analysis utilized the embedded intercept to identify horizontal pleiotropy and generated accurate estimates following adjustment for pleiotropy effects. The MR-PRESSO global test can estimate the level of pleiotropy caused by SNP heterogeneity after using MR-PRESSO to identify and adjust for potential outliers.^17^

FEV1 and FVC were considered outcomes, with β being utilized to evaluate the impact of T2DM and associated glycemic indicators on lung function. All evaluations were conducted considering both possibilities. For this analysis, the *R* packages TwoSampleMR and MR-PRESSO in R 4.3.2 were used.^22^

### Mediation analysis

MR was performed on two samples to assess the overall impact of T2DM on lung function. The total influence of a given exposure on an outcome can be analyzed by considering both indirect and direct consequences.^23^ MVMR was used to determine the direct impact of exposure on outcomes, including T2DM, fasting insulin, fasting glucose, and two-hour glucose while accounting for the mediators. The mediated effect, known as the indirect effect, was calculated by multiplying β1 and β2. Here, β1 represents the effect of exposure on mediators, while β2 represents the effect of mediator k on outcome after adjusting for genetically determined glycemic traits. The proportion of the mediation Effect (E%) was estimated using the following equation^24^^,^^25^^:^ $E\left(\%\right)=\frac{\sum_{k=1}^{k}\beta 1\times \beta 2k}{\sum_{k=1}^{k}\beta 3+\beta 1\times \beta 2k}$; β3 represents the impact of glycemic characteristics on lung function after accounting for potential mediators determined by genetics.

## Results

### Selection of IVs

Independent SNPs were identified by screening for SNPs with LD using thresholds of p <  5×10^−8^. Next, palindromic SNPs were excluded, and outlier SNPs were removed via MR-PRESSO analysis.

### Causal association of glycemic traits with lung function via univariable MR

**Fig 2:**
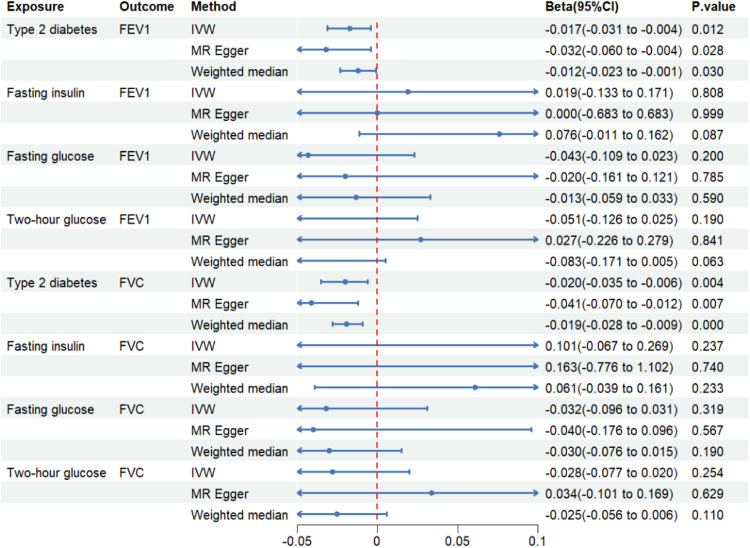
Causal effect of glycemic traits on lung function. The blue point indicates the effect (Beta). FEV1, Forced Expiratory Volume in 1s; FVC, Forced Vital Capacity; IVW, Inverse Variance Weighting; MD, Mendelian Randomization.

A negative association between genetically predicted T2DM and FEV1 (IVW: Beta -0.017 [-0.031, -0.004], p = 0.012) and FVC (IVW: -0.020 [-0.035, -0.006], p = 0.004) was observed. These findings are consistent with those of previous studies. However, based on the IVW evaluation, genetically estimated fasting insulin, fasting glucose, and two-hour glucose did not show significant associations with lung function. Fig. 2, Supplemental Table 2 displays the outcomes achieved using the various methods. Consistent results were obtained using the MR-PRESSO method.

### Causal association of confounders with lung function via univariable MR

**Fig. 3:**
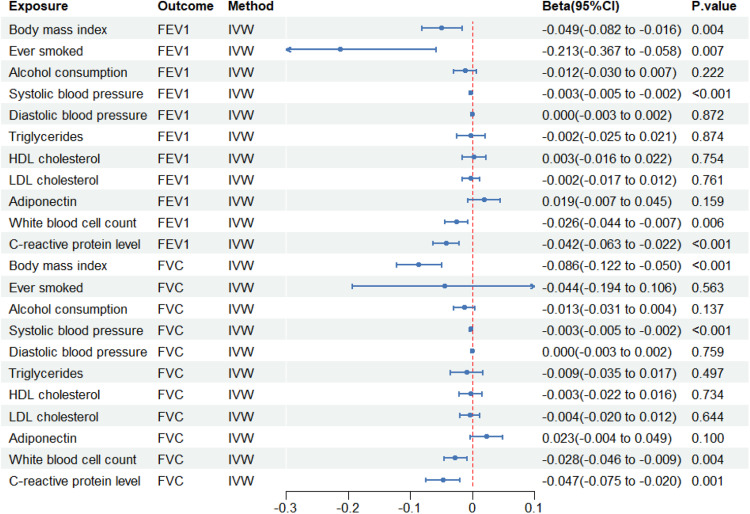
Causal effect of confounders on lung function. The blue point indicates the effect (Beta). LDL, Low-Density Lipoprotein; HDL, High-Density Lipoprotein; FEV1, Forced Expiratory Volume in 1s; FVC, Forced Vital Capacity; IVW, Inverse Variance Weighting.

The study revealed that predicted Body Mass Index (BMI), smoking status, Systolic Blood Pressure (SBP), white blood cell count, and C-Reactive Protein (CRP) levels were all associated with reduced FEV1. Furthermore, genetic predictions of BMI, SBP, white blood cell count, and CRP levels were linked to a reduction of FVC. Conversely, IVW estimation indicated that genetically forecasted alcohol intake, diastolic blood pressure, triglyceride, High-Density Lipoprotein (HDL) cholesterol, Low-Density Lipoprotein (LDL) cholesterol, and adiponectin levels were not linked to pulmonary function. Fig. 3 and Supplementary Table 3 show the outcomes achieved using the various methods.

### Causal association of type 2 diabetes with confounders via univariable MR

**Fig 4:**
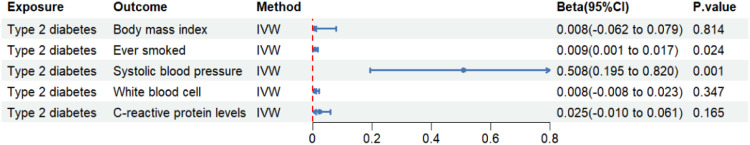
Casual effect of T2DM on body mass index, smoking status, systolic blood pressure, white blood cell count, and C-reactive protein level. The blue point indicates the effect (Beta). IVW, Inverse Variance Weighting.

The findings indicated a positive correlation between the genetically predicted risk of T2DM and higher rates of smoking (IVW Beta = 0.009 [0.001, 0.017], p = 0.024) and elevated SBP (IVW Beta = 0.508 [0.195, 0.820], p = 0.001). The results of the various methods are shown in Fig. 4 and Table 1. The results of MR-PRESSO were similar.

**Table 1:** Result and sensitivity analyses for the associations of genetically predicted factors with mediators.

Exposure	Outcome	MR					Heterogeneity			Pleiotopy	
		Method	Beta	(95%CI)	SE	p-value	Method	Cochran's *Q*	p-value	MR-Egger intercept	p-value
**Type 2 diabetes**	Body mass index	IVW	0.008	(-0.062, 0.079)	0.036	0.814	IVW	1312.510	**<0.001**	0.007	0.345
	Ever smoked	IVW	0.009	(0.001, 0.017)	0.004	**0.024**	IVW	209.973	**<0.001**	-0.001	0.893
	Systolic blood pressure	IVW	0.508	(0.195, 0.820)	0.159	**0.001**	IVW	474.929	**<0.001**	0.963	0.963
	White blood cell count	IVW	0.008	(-0.008, 0.023)	0.008	0.347	IVW	44.045	0.554	0.000	0.825
	C-reactive protein levels	IVW	0.025	(-0.010, 0.061)	0.018	0.165	IVW	379.089	**<0.001**	-0.002	0.397

CI, Confidence Interval; SE, Standard Error; MR, Mendelian Randomization.

### Mediating effect and proportion by smoking status and systolic blood pressure

The direct effects in the multivariable MR analysis of T2DM-smoking-FEV1, T2DM-SBP-FEV1, and T2DM-SBP-FVC were reduced to Beta -0.012 (95% CI -0.026, -0.002; p = 0.100), Beta -0.025 (95% CI -0.041, -0.008, p = 0.003), and Beta -0.003 (95% CI -0.049, -0.016, p < 0.001), respectively (Table 2). The proportions mediated by these mediators were 0.302, 0.412, and 0.418, respectively, and the proportion mediated by SBP was 0.504. After adjusting for both smoking and SBP, the direct effect was Beta -0.025. The proportion mediated by smoking and SBP was 0.43.

**Table 2:** Multivariate MR analysis of the direct effect of Systolic blood pressure and smoking on lung function.

Exposure/Outcome	Adjusted Factors	Beta	(95%CI)	p-value	Mediation Effect
Type 2 diabetes/FEV1	None	-0.017	(-0.031, -0.004)	0.012	
Type 2 diabetes/FEV1	Smoking	-0.012	(-0.026, -0.002)	0.100	0.302
Type 2 diabetes/FEV1	Systolic blood pressure	-0.025	(-0.041, -0.008)	0.003	0.412
Type 2 diabetes/FEV1	Smoking, Systolic blood pressure	-0.025	(-0.071, 0.102)	0.003	0.418
Type 2 diabetes/FVC	None	-0.020	(-0.035, -0.006)	0.004	
Type 2 diabetes/FVC	Systolic blood pressure	-0.033	(-0.049, -0.016)	<0.001	0.594

CI, Confidence Interval.

## Discussion

In this study, a genetic predisposition to T2DM, smoking status, and high SBP were associated with lung function reduction. The individual proportions mediated by smoking and SBP on FEV1 were 0.302 and 0.412, respectively, whereas the proportion mediated by SBP on FVC was 0.594. The proportion that was mediated by smoking and SBP together was 0.418. The MR study demonstrated that those diagnosed with T2DM are more likely to have reduced lung function.

Glycemic traits refer to a range of measurable biological markers associated with blood glucose levels and how the body processes glucose. The Meta-Analyses of Glucose and Insulin-related Traits Consortium (MAGIC) previously analyzed GWAS data on glycemic traits in non-diabetic individuals, identifying 16 loci associated with fasting glucose, two with fasting insulin, and five with post-challenge Glucose (2hGlu) concentrations.^26^ Fasting glucose and two-hour glucose represent blood glucose levels. The authors also used the fasting insulin level as an exposure factor, which provides a proxy for the degree of insulin resistance.^27^ Lung function typically refers to a set of physiological measurements that assess respiratory health and the efficiency of the lungs. It is commonly assessed by FEV1 and FVC.^28^

The present findings align with previous research showing reduced FVC and FEV1 in adults with diabetes compared with those without diabetes.^29^^,^^30^ Nevertheless, these results were largely derived from observational research, with the potential for numerous confounding factors, such as data collection methods, genetics specific to certain populations, and exposure to environmental factors. Hence, in this study, MR was performed to investigate the relationship between lung function and glycemic traits. MR-Egger regression was used to assess the potential pleiotropic effects of SNPs chosen as IVs, offering insights into whether horizontal pleiotropy affects the analysis. Horizontal pleiotropy was not observed in any of the analyses.

This finding is consistent with that of a previous study that was less susceptible to confusion bias. Autopsies of individuals with diabetes revealed an increase in the thickness of the alveolar walls and minor blood vessels.^31^ These changes could be the cause of restriction in lung expansion and a decrease in ventilatory capacity. Klein et al. demonstrated that the lungs in individuals with diabetes could experience microangiopathy.^32^ Individuals with diabetes experience reduced lung capacity compared with those without diabetes, irrespective of additional risk factors. Glycemic control and the length of time a person has diabetes are the primary factors that pose the greatest risk.^7^

The present study showed that smoking was associated with impaired lung function, as indicated by decreased FEV1. Smoking is a harmful agent that contributes to many health problems worldwide, including lung disease.^33^ Smoking during adulthood is classically associated with an accelerated decrease in FEV1.^34^ A previous study demonstrated that, between the ages of 43- and 60–64 years, the decrease in FEV1 was accelerated by smoking; however, smoking did not influence the decrease in FVC.^35^ Another study indicated that the annual rate of FEV1 loss was greater in continuing smokers, by more than 10 mL/year, than in non-smokers.^36^ In summary, smokers exhibit changes in lung morphology, inflammation, and function.^37^ Smoking control is a pivotal health concern, and there is an urgent need to correct alterations in the risk perception of smoking and to cease smoking early.

This study revealed an inverse relationship between SBP and lung function. Previous research found that decreased FVC was linked to hypertension, and this connection remained consistent regardless of weight and age.^38^ Another study^39^ reported a robust negative correlation between FVC and the likelihood of high blood pressure. Additionally, previous research found an inverse correlation between lung function and blood pressure among Chinese individuals, although the correlation was minimal.^40^ In contrast, another study revealed a significant correlation among hypertension, beta-blocker use, and decreased pulmonary function in German adults.^41^ A recent study from Korea suggested a connection between high blood pressure and an increased decline in FVC and that medications for hypertension could slow this decline in asymptomatic individuals.^42^ However, the mechanism underlying the relationship between lung function and hypertension requires further investigation.

The present study benefited from the use of a stronger MR approach that enforces a more rigorous IV selection process as well as the implementation of strict criteria for IV selection to enhance statistical power. In addition, this study used GWAS datasets from large European populations for both exposure and outcomes, which enhanced the credibility of the results.

Nevertheless, this study has some limitations. First, while smoking status is a significant environmental factor and a possible confounding variable in the association between blood glucose levels and respiratory illnesses in individuals with compromised lung function, the results were not corrected for smoking habits. Second, due to GWAS limitations, the authors used fasting insulin levels as a proxy for insulin resistance. Although this indicator can indicate insulin resistance to some extent, it isn't the gold standard.^43^ Furthermore, the association between glycemic traits and lung function was limited to the European population, suggesting possible selection biases and further investigation is needed to determine whether the results can be generalized to populations of other races. In addition, in the analysis, there were some differences in composition. Due to the utilization of GWAS data, it was not feasible to explore potential nonlinear associations or stratification effects that are dependent on age, health condition, or sex.

## Conclusions

The study found a connection between the predicted genetic risk of T2DM, smoking status, SBP, and reduction in FEV1 and FVC. Thus, strengthening blood pressure and blood sugar control, and changing unhealthy lifestyles may serve as potential intervention targets for preventing impaired lung function.

## Ethics approval

As this study was based on a re-analysis of previously conducted and published GWASs data, there is no requirement for further ethical approval.

## Consent to publish

Not applicable.

## Consent to participate

Not applicable.

## Availability of data and materials

All data relevant to the study are included in the article or uploaded as supplementary information.

## Funding

This work was supported by 10.13039/501100013290National Key Research and Development Program of China (grant number: 2018YFC1313902).

## CRediT authorship contribution statement

**Shuo Xie:** Conceptualization, Methodology, Formal analysis, Writing – original draft. **Liping Yu:** Supervision. **Lulu Song:** Supervision. **Fei Chen:** Supervision. **Wanlu Ma:** Supervision. **Yifan He:** . **Xiaoping Chen:** Supervision. **Ying Yang:** Writing – review & editing. **Bo Zhang:** Conceptualization, Supervision, Funding acquisition.

## Conflicts of interest

The authors declare no conflicts of interest.

## Appendix Supplementary materials

Image, application 1
