# Supplementary material for: New evidence for the effect of type 2 diabetes and glycemic traits on lung function: a Mendelian randomization and mediation analysis
Source: Clinics (Sao Paulo). 2025 Jul 15;80:100693. doi: 10.1016/j.clinsp.2025.100693 (PMC12281521; doi:10.1016/j.clinsp.2025.100693)
Supplement: Supplementary file 1 [file mmc1.doc]

CLINICS-D-24-00539_Supplementary Materials

**Supplementary Table 1** Information of included studies and consortia.

| **Exposure/Covariate/Outcome** | **Traits** | **Accession Number^a^** | **Consortium/First author** | **Sample Size** |
| --- | --- | --- | --- | --- |
| Exposures | Type 2 diabetes | finn-b-E4_DM2 | FinnGen Biobank | 215654 |
|  | Fasting insulin | ieu-b-116 | MAGIC | 108557 |
|  | Fasting glucose | ieu-b-114 | MAGIC | 133010 |
|  | Two-hour glucose | ebi-a-GCST90002227 | Chen J | 63396 |
| Covariate | Body mass index | ieu-a-2 | GIANT | 339224 |
|  | Ever smoked | ieu-b-4858 | Within family GWAS consortium | 99996 |
|  | Alcohol consumption | ieu-b-4834 | Within family GWAS consortium | 83626 |
|  | Physical activity | ieu-b-4860 | Within family GWAS consortium | 78007 |
|  | Systolic blood pressure | ieu-b-38 | International Consortium of Blood Pressure | 757601 |
|  | Diastolic blood pressure | ieu-b-39 | International Consortium of Blood Pressure | 757601 |
|  | Triglycerides | ieu-a-302 | GLGC | 177861 |
|  | HDL cholesterol | ieu-a-299 | GLGC | 187167 |
|  | LDL cholesterol | ieu-a-300 | GLGC | 173082 |
|  | Adiponectin | ieu-a-1 | ADIPOGen | 39883 |
|  | white blood cell | ieu-b-30 | Blood Cell Consortium | 563946 |
|  | C-reactive protein | ieu-b-35 | Ligthart, S | 204,402 |
| Outcome | FEV1 | ukb-b-19657 | MRC IEU | 421986 |
|  | FVC | ukb-b-7953 | MRC IEU | 421986 |

^a^ GWAS summary datasets of glycemic traits are from the MRC IEU OpenGWAS database (https://gwas.mrcieu.ac.uk/).

**Supplementary Table 2** Result and sensitivity analyses for the associations of genetically predicted type 2 diabetes and glycemic traits with lung function.

| **Exposure** | **Outcome** | **Inverse variance weighted** | | | | | | **Weighted median** | | | | **MR-Egger estimate** | | | | **MR-Egger intercept** | | | **MR-PRESSO** | | | | | |
| --- | --- | --- | --- | --- | --- | --- | --- | --- | --- | --- | --- | --- | --- | --- | --- | --- | --- | --- | --- | --- | --- | --- | --- | --- |
|  |  | ***Q*** | **Cochran's *Q* test, p-value** | **Beta** | **(95%CI)** | **SE** | **p-value** | **Beta** | **(95% CI)** | **SE** | **p-value** | **Beta** | **(95% CI)** | **SE** | **p-value** | **Intercept** | **SE** | **p-value** | **Outliers** | **Global test, p-value** | **Beta** | **SE** | **p-value** | **Distortion test, p-value** |
| Type 2 diabetes | FEV1 | 364.800 | **<0.001** | -0.017 | (-0.031, -0.004) | 0.007 | **0.012** | -0.012 | (-0.023, -0.001) | 0.005 | **0.030** | -0.032 | (-0.045, -0.004) | 0.014 | 0.028 | 0.002 | 0.001 | 0.240 | 8 | **<0.001** | -0.010 | 0.004 | **0.031** | 0.009 |
| Fasting insulin |  | 242.249 | **<0.001** | 0.019 | (-0.133, 0.171) | 0.077 | 0.808 | 0.076 | (-0.011, 0.162) | 0.044 | 0.087 | -0.001 | (-0.683, 0.683) | 0.348 | 0.999 | 0.000 | 0.005 | 0.956 | 3 | **<0.001** | 0.034 | 0.039 | 0.408 | 0.001 |
| Fasting glucose |  | 170.327 | **<0.001** | -0.043 | (-0.109, 0.023) | 0.034 | 0.200 | -0.013 | (-0.059, 0.033) | 0.023 | 0.590 | -0.020 | (-0.161, 0.121) | 0.072 | 0.785 | -0.001 | 0.002 | 0.715 | 5 | **<0.001** | -0.014 | 0.023 | 0.541 | 0.020 |
| Two-hour glucose |  | 10.739 | 0.150 | -0.051 | (-0.126, 0.025) | 0.039 | 0.190 | -0.083 | (-0.171, 0.004) | 0.045 | 0.063 | 0.027 | (-0.226, 0.279) | 0.129 | 0.841 | -0.002 | 0.003 | 0.549 | 4 | **<0.001** | -0.007 | 0.013 | 0.576 | <0.001 |
| Type 2 diabetes | FVC | 446.895 | **<0.001** | -0.020 | (-0.035, -0.006) | 0.007 | **0.004** | -0.019 | (-0.028, -0.009) | 0.005 | **<0.001** | -0.041 | (-0.070, -0.012) | 0.015 | **0.007** | 0.002 | 0.002 | 0.112 | 10 | **<0.001** | -0.015 | 0.004 | 0.001 | 0.020 |
| Fasting insulin |  | 103.966 | **<0.001** | 0.101 | (-0.067, 1.106) | 0.086 | **0.237** | 0.061 | (-0.039, -0.161) | 0.051 | 0.233 | 0.163 | (-0.776, 1.102) | 0.479 | 0.740 | -0.001 | 0.008 | 0.898 | 4 | **<0.001** | 0.066 | 0.042 | 0.152 | 0.066 |
| Fasting glucose |  | 176.613 | **<0.001** | -0.032 | (-0.096, 0.031) | 0.032 | 0.319 | -0.030 | (-0.076, 0.015) | 0.023 | 0.190 | -0.040 | (-0.176, 0.096) | 0.069 | 0.567 | 0.000 | 0.002 | 0.898 | 6 | **<0.001** | -0.033 | 0.026 | 0.211 | 0.973 |
| Two-hour glucose |  | 151.725 | **<0.001** | -0.028 | (-0.077, 0.020) | 0.025 | 0.254 | -0.025 | (-0.056, 0.006) | 0.016 | 0.110 | 0.034 | (-0.101, 0.169) | 0.069 | 0.629 | -0.005 | 0.005 | 0.349 | 6 | **<0.001** | -0.026 | 0.014 | 0.104 | 0.492 |

CI, Confidence Interval; SE, Standard Error; MR, Mendelian Randomization; MR-PRESSO, Mendelian Randomization-Pleiotropy Residual Sum and outlier.

* Outlier-corrected estimate.

**Supplementary Table 3** Result and sensitivity analyses for the associations of genetically predicted behavior factor, metabolic traits, inflammatory traits with lung function.

| **Exposure** | **Outcome** | **MR** | | | | **Heterogeneity** | | | **Pleiotopy** | |
| --- | --- | --- | --- | --- | --- | --- | --- | --- | --- | --- |
|  |  | **Method** | **Beta (95%CI)** | **SE** | **p-value** | **Method** | **Cochran's Q** | **p-value** | **MR-Egger intercept** | **p-value** |
| Body mass index | FEV1 | IVW | -0.049 (-0.082, -0.016) | 0.017 | **0.004** | IVW | 351.510 | **<0.001** | -0.001 | 0.550 |
| Ever smoked |  | IVW | -0.213 (-0.367, -0.058) | 0.079 | **0.007** | IVW | 10.208 | 0.070 | -0.006 | 0.450 |
| Alcohol consumption |  | IVW | -0.012 (-0.030, 0.007) | 0.009 | 0.222 | IVW | 2.286 | 0.808 | -0.010 | 0.621 |
| Systolic blood pressure |  | IVW | -0.003 (-0.005, -0.002) | 0.001 | **<0.001** | IVW | 2364.298 | **<0.001** | -0.001 | 0.103 |
| Diastolic blood pressure |  | IVW | -0.001 (-0.003, 0.002) | 0.001 | 0.872 | IVW | 2979.342 | **<0.001** | 0.001 | 0.191 |
| Triglycerides |  | IVW | -0.002 (-0.025, 0.021) | 0.012 | 0.874 | IVW | 253.813 | **<0.001** | 0.000 | 0.632 |
| HDL cholesterol |  | IVW | 0.003 (-0.016, 0.022) | 0.010 | 0.754 | IVW | 329.411 | **<0.001** | 0.000 | 0.833 |
| LDL cholesterol |  | IVW | -0.002 (-0.017, 0.012) | 0.007 | 0.761 | IVW | 290.480 | **<0.001** | -0.001 | 0.418 |
| Adiponectin |  | IVW | 0.019 (-0.007, 0.045) | 0.013 | 0.159 | IVW | 31.486 | **0.003** | 0.003 | 0.058 |
| White blood cell count |  | IVW | -0.026 (-0.044, -0.007) | 0.009 | **0.006** | IVW | 2563.474 | **<0.001** | -0.001 | 0.131 |
| C-reactive protein |  | IVW | -0.042 (-0.063, -0.022) | 0.010 | **<0.001** | IVW | 603.485 | **<0.001** | -0.002 | 0.018 |
| Body mass index | FVC | IVW | -0.086 (-0.122, -0.050) | 0.018 | **<0.001** | IVW | 458.526 | **<0.001** | 0.000 | 0.801 |
| Ever smoked |  | IVW | -0.044 (-0.194, 0.106) | 0.076 | 0.563 | IVW | 10.724 | 0.057 | -0.008 | 0.309 |
| Alcohol consumption |  | IVW | -0.013 (-0.031, 0.004) | 0.009 | 0.137 | IVW | 1.945 | 0.857 | -0.008 | 0.679 |
| Systolic blood pressure |  | IVW | -0.003 (-0.005, 0.002) | 0.001 | **<0.001** | IVW | 2754.289 | **<0.001** | -0.002 | 0.006 |
| Diastolic blood pressure |  | IVW | 0.000 (-0.03, 0.002) | 0.001 | 0.759 | IVW | 3397.294 | **<0.001** | 0.001 | 0.634 |
| Triglycerides |  | IVW | -0.009 (-0.035, 0.016) | 0.013 | 0.497 | IVW | 363.356 | **<0.001** | -0.001 | 0.184 |
| HDL cholesterol |  | IVW | -0.003 (-0.022, 0.016) | 0.010 | 0.734 | IVW | 373.847 | **<0.001** | 0.000 | 0.581 |
| LDL cholesterol |  | IVW | -0.004 (-0.020, 0.012) | 0.008 | 0.644 | IVW | 401.257 | **<0.001** | -0.001 | 0.385 |
| Adiponectin |  | IVW | 0.023 (-0.004, 0.049) | 0.014 | 0.100 | IVW | 37.015 | **<0.001** | 0.002 | 0.085 |
| white blood cell count |  | IVW | -0.028 (-0.046, -0.009) | 0.009 | **0.004** | IVW | 2921.793 | **<0.001** | -0.001 | 0.238 |
| C-reactive protein levels |  | IVW | -0.047 (-0.075, -0.020) | 0.014 | **0.001** | IVW | 555.617 | **<0.001** | -0.003 | 0.004 |

CI, Confidence Interval; SE, Standard Error; MR, Mendelian Randomization.
